# von Willebrand factor rescued by *miR-24* inhibition facilitates the proliferation and migration of osteosarcoma cells *in vitro*

**DOI:** 10.1042/BSR20180372

**Published:** 2018-11-16

**Authors:** Ling Liu, Jun Pan, Huan Wang, Zhenni Ma, Jie Yin, Feng Yuan, Quanwen Yuan, Lu Zhou, Xiaofeng Liu, Yu Zhang, Zhaohua Bao, Huilin Yang, Jing Ling

**Affiliations:** 1 Department of Orthopedics, Clinical Medical Research Center of Jiangsu Province, The First Affiliated Hospital of Soochow University, Suzhou 215006, China; 2 Department of Hematology and Oncology, Children’s Hospital of Soochow University, Suzhou 215003, China; 3 Jiangsu Institute of Hematology, Key Laboratory of Thrombosis and Hemostasis of Ministry of Health, The First Affiliated Hospital of Soochow University, Suzhou 215006, China; 4 Collaborative Innovation Center of Hematology, Soochow University, Suzhou 215006, China; 5 Department of Orthopedics, The Affiliated Hospital of Xuzhou Medical University, Xuzhou 221003, China; 6 Department of Orthopedics, Children’s Hospital of Soochow University, Suzhou 215003, China; 7 Department of Hematology, Affiliated Hospital of Nantong University, Nantong, 226000 China; 8 Department of Orthopedics, The Friendship Hospital of Ili Kazakh Autonomous Prefecture, Yining 835000, China; 9 Department of Orthopedics, Jiangsu Province Hospital, The First Affiliated Hospital of Nanjing Medical University, Nanjing 210029, China

**Keywords:** miR-24, migration, osteosarcoma, proliferation, von Willebrand factor

## Abstract

von Willebrand factor (vWF) is a major procoagulant molecule that was shown to differentiate between metastatic and primary osteosarcoma (OS) tissues and associated with increased metastasis. However, its functional role in OS progression has been unclear yet. The expression profile of vWF and *miR-24* in human OS tissues was characterized using immunofluorescence labeling and quantitative real-time PCR analysis. The interaction between *miR-24* and vWF was identified by dual luciferase reporter assay. The effects of vWF and *miR-24* on OS cells were assessed by cell proliferation, colony formation, and migration. The clinical significance of *miR-24* in OS patients was analyzed using Kaplan–Meier analyses and Pearson’s Chi-squared test. Here, we reported that the expression of vWF was significantly increased, but *miR-24* was significantly decreased in OS tissues (*n*=84). vWF was further validated as the target of *miR-24* in MG-63 and U2OS cells. *miR-24* obviously suppressed the proliferation and migration of MG-63 and U2OS cells. However, the migration-inhibiting activity of *miR-24* was predominantly attenuated by vWF overexpression. Clinically, low *miR-24* expression in human OS tissues was significantly associated with tumor metastasis and predicted a poor survival in OS patients. This work demonstrated that vWF, as a downstream effector of *miR-24*, played an important role in controlling OS cell progression. Target *miR-24* or vWF, therefore, promises to be an effective biological target for OS treatment.

## Introduction

Osteosarcoma (OS) is a malignant bone cancer with high mortality in children and young adults worldwide [1]. Although great progress has been made in both clinical diagnosis and treatment for OS during the recent decade, high distant metastasis and recurrence rate lead to the worse long-term survival and prognosis in OS patients [2]. As complicated pathologies of OS, the underlying mechanism of OS initiation and progression remains unclear so far. Therefore, a good understanding of the precise mechanism associated with OS is urgently required for developing effective therapeutic approaches.

von Willebrand factor (vWF) is a complex multimeric plasma glycoprotein and its important role in hemostasis has been well documented [3]. Emerging roles of vWF involved in cancer cell biology, especially in tumor metastasis, were also identified in many types of human cancers [4–6]. Of note, vWF was suggested as the important mediator of platelet–tumor cells interactions, thereby promoting metastasis [6]. And this conclusion was further confirmed by the finds that vWF inhibition effectively decreased tumor metastasis in murine and reduced the adhesion of colon cancer cells to endothelial cells (ECs) [7,8]. Moreover, the high levels of plasma vWF were detected in patients with glioblastoma, acute lymphoblastic leukemia, colorectal cancer, urinary bladder cancer, and other types of cancers [9–12]. It has been reported that vWF is involved in cell death, for example, vWF would be able to control bone cancer cells apoptosis by controlling the binding of osteoprotegerin to TRAIL [13]. In addition, vWF was also shown to be highly expressed in metastatic OS tissues compared with primary OS tissues, suggesting a potential role of vWF dysregulation in OS metastasis [14]. However, the functional involvement of vWF in OS and its mechanism remain unknown.

Accumulating studies reported that miRNAs were abnormally expressed in cancer cells and played a critical role in modulating cancer cell biology, including proliferation, migration, invasion, and apoptosis [15]. As non-coding RNA transcript, miRNAs could transcriptionally or post-transcriptionally regulate gene expression through binding to the 3′-UTR of target mRNA [16, 17]. It has been widely identified that *miR-24* mediated cell proliferation and death [18]. Several miRNAs were identified to be related to malignant biological properties of OS, including *miR-24*. Originally, *miR-24* was found to down-regulate in OS cells, and the forced *miR-24* inhibited the proliferation of OS cells by targetting lysophosphatidic Acid Acyltransferase β (LPAATβ) [19]. Subsequently, increasing evidence demonstrated that *miR-24* functioned as a tumor suppressor in OS progression [20]. Hence, it was reasonable to propose that *miR-24* alternation has important pathological significance in OS. It experimentally revealed that *miR-24* interacted with the 3′-UTR of vWF [21]. Considering their important role in OS, we therefore inferred that targetting vWF with *miR-24* was involved in the progression of OS. The aim of the present study was to characterize the functional role of vWF in OS progression and to examine the existence of *miR-24*/vWF axis in the oncogenesis and progression of OS.

## Materials and methods

### Patients and specimens

The present study was approved by the Ethics Committee of the related institutions, and written informed consents were obtained from all patients enrolled in our study. A total of 84 human OS tissues and adjacent non-cancerous tissues were collected from the patients which underwent surgical resection in The First Affiliated Hospital of Soochow University, Children’s Hospital of Soochow University, The Affiliated Hospital of Xuzhou Medical University, The Friendship Hospital of Ili Kazakh Autonomous Prefecture, Jiangsu Province Hospital, The First Affiliated Hospital of Nanjing Medical University, and all patients did not receive any chemotherapy or radiation before surgery. Histological diagnosis and grading were performed by two independent pathologists. The fresh specimens were stored at −80°C for .

### Immunofluorescence labeling assays

The cell glass slides and tissue cryostat sections at 5 μm of OS tissues or adjacent non-cancerous tissues were used for the immunofluorescence labeling of vWF as previously described [19]. Briefly, the sections were fixed with methyl alcohol (95%) for 5 min, then incubated with primary antibodies against vWF (Dako, Glostrup, Denmark) and PECAM (Abcam, U.K.) overnight at 4°C. The sections were washed in PBS for 3 times × 5 min and incubated with the Alexa fluor 488, 555, or 647 labeled secondary antibody (Thermo Scientific, Waltham, MA, U.S.A.) for 1 h. After DAPI staining, the images were taken under an inverted microscope (Olympus, Tokyo, Japan).

### Cell culture and transfection

Human OS cell lines MG-63, U2OS, and KHOS were purchased from the American Type Culture Collection (ATCC, Manassas, VA, U.S.A.). OS cell lines were cultured in RPMI-1640 medium (HyClone, Logan, UT, U.S.A.) containing 10% fetal bovine serum (FBS, Gibco, Grand Island, NY, U.S.A.), 100 IU/ml penicillin and 100 mg/ml streptomycin. All cells were cultured in a humidified atmosphere containing 5% CO_2_ at 37°C.

In the present study, *miR-24* mimics/inhibitor (Guangzhou RiboBio Co., Ltd., Guangzhou, China) and pcDNA-vWF (Shanghai GenePharma Co., Ltd., Shanghai, China) were used to respectively modulate the intracellular level of *miR-24* and vWF. For transfection, MG-63, U2OS cells were cultured in six-well plates (1 × 10^6^ cells/well) and then transfected with *miR-24* mimics (50 nM), inhibitor (30 nM), pcDNA-vWF (2 μg), or corresponding negative control using Lipofectamine 2000 (Invitrogen, Waltham, MA, U.S.A.) according to the manufacturer’s protocol. After 48 h of the transfection, the cells were collected for the following analysis.

### Quantitative real-time PCR

Total RNA was extracted from the tissue specimens and cells using TRIzol reagent (Invitrogen, Waltham, MA, U.S.A.) following the manufacturer’s protocol. The purified RNA was quantitated using a NanoDrop 2000 (Thermo Scientific, Waltham, MA, U.S.A.). For analysis of *miR-24*, total RNA (2.5 μg) was reverse transcribed into cDNA using miRNA First-Strand cDNA Synthesis Kit (Thermo Scientific, Waltham, MA, U.S.A.), and quantitative PCR was then performed with SYBR Green miRNA qRT-PCR kit (Thermo Scientific, Waltham, MA, U.S.A.) and specific primers according to the manufacturer’s protocol. For vWF mRNA measurement, reverse transcript was performed using Reverse Transcription Kit (Takara, Dalian, China). And quantitative PCR was carried out using SYBR Green qPCR Master Mix (Applied Biosystems, Foster City, CA, U.S.A.). The relative abundance of *miR-24* and vWF, as determined by the 2^−ΔΔ*C*^_T_ method, was normalized to U6 and GAPDH, respectively. The gene-specific primers used in the present study were synthesized by Shanghai Sangon Biotech (Shanghai, China). The paired primer sequences were listed: *miR-24*, (forward) 5′-ACACTCCAGCTGGGTGGCTCAGTTCAGCAG-3′, (reverse) 5′-CTCAACTGGTGTCGTGGAGTCGGCAATTCAG-3′; U6, (forward) 5′- CTCGCTTCGGCAGCACA-3′, (reverse) 5′- AACGCTTCACGAATTTGCGT-3′; vWF, (forward) 5′-CGGCTTGCACCATTCAGCTA-3′, (reverse) 5′-TGCAGAAGTGAGTATCACAGCCATC-3′; GAPDH, (forward) 5′-GGCTGTTGTCATACTTCTCATGG-3′, (reverse) 5′-GGCTGTTGTCATACTTCTCATGG-3′.

### Western blotting

Protein samples were extracted from the cultured cells using RIPA buffer (Roche, Pleasanton, CA, U.S.A.) supplemented with a protease inhibitor (cocktail) according to the manufacturer’s protocol. The concentration of total protein was determined by BCA Kit (Thermo Scientific, Waltham, MA, U.S.A.). Equal protein (25 μg) of each sample was separated by SDS/PAGE and then transferred to PVDF membrane (Millipore, Danvers, MA, U.S.A.). The PVDF membrane was blocked in 5% skim milk for 1 h and incubated with primary antibodies against vWF (dilution of 1:500, Cell Signaling Technology, Danvers, MA, U.S.A.) and β-actin (dilution of 1:1000, Cell Signaling Technology, Danvers, MA, U.S.A.) at 4°C overnight. The PVDF membrane was washed with TBS/Tween-20 and incubated with HRP-conjugated secondary antibodies for 1 h at room temperature. The Immunoreactive bands were visualized by enhanced chemiluminescence substrate (Millipore, Boston, MA, U.S.A.).

### Luciferase reporter assay

According to the putative binding site of *miR-24* in the 3′-UTR of vWF (wild type), the site was mutated from GAGCC to ACAUU to generate the mutated vWF 3′-UTR using QuickChange Site-Directed Mutagenesis Kit (Stratagene). The wild type and mutated vWF 3′-UTR was cloned into the pmiR-GLO reporter vector (Promega Corporation, Madison, WI, U.S.A.), respectively. MG-63 cells (5 × 10^4^ cells/well) were cultured in 24-well plates for 24 h, and co-transfected with luciferase reporter plasmid containing vWF 3′-UTR (wild type/mutated) and *miR-24* mimic/miR-C using Lipofectamine 2000 (Invitrogen) according to the manufacturer’s protocol. After 48 h of transfection, luciferase activity in MG-63 cells was detected using the Dual-Luciferase Reporter assay system (Promega, Madison, WI, U.S.A.) following the manufacturer’s instructions.

### Cell proliferation and colony formation assays

Cell Counting Kit-8 (CCK-8) assay was used to assess the cell proliferation of MG-63 and U2OS cells. Cells (5 × 10^3^ cells/well) were cultured in 96-well plates for 24 h, and then incubated with CCK-8 solution (10 μl/well) (Dojindo Laboratories, Kumamoto, Japan) for 2 h at 37°C. The absorbance of each well was measured at 450 nm using Microplate Reader (Bio-Rad, Foster, California, U.S.A.). In colony formation assay, Cells (1 × 10^3^ cells/well) were cultured in six-well plates for 10 days. The formed colonies were washed with PBS and fixed in ethanol for 20 min. The cloning capability of cells was evaluated by means of crystal violet staining. The fixed colonies were stained with 0.1% crystal violet (Sigma–Aldrich, St. Louis, MO, U.S.A.) for 10 min. The stained colonies were counted using an inverted microscope (Olympus, Tokyo, Japan).

### Cell apoptosis

OS cells apoptosis were assessed by using the fluorescein isothiocyanate annexin V Apoptosis Detection Kit (Beyotime, Shanghai, China). Briefly, cultured cells were collected, washed with cold PBS, and resuspended in 1× binding buffer (0.01 mmol/l HEPES [pH 7.4], 0.14 mol/l NaCl, and 2.5 mmol/l CaCl_2_) at a concentration of 2 × 10^6^ cells/ml. One hundred microliters of cell solution were transferred into a 5-ml culture tube, and 5 μl of annexin V was added. After gentle mixing and incubation for 15 min at room temperature in the dark, 400 μl of the 1× binding buffer was added, and cells were analyzed by flow cytometry.

### Wound-healing assays

Cells (1 × 10^6^ cells/well) were cultured in six-well plates till 90% confluence was reached. Artificial wounds were then induced by a sterile plastic tip, and the cells were washed with serum-free medium. After another 48 h of culture, the images of wound areas were recorded using an inverted microscope (Olympus, Tokyo, Japan). The migration efficiency was calculated by the following formula: migration efficiency (%) = (wound area [0 h] − wound area [48 h])/wound area (0 h) × 100%.

### Statistical analysis

Data from at least three independent sample replicates were presented as the mean ± S.D. All statistical analysis was conducted using SPSS 21.0 statistical software. A value of *P* < 0.05 was considered as statistically significant. The statistical differences between two groups were analyzed using Student’s *t* test. The relative expressions of *miR-24* in human OS tissues and adjacent non-cancerous tissues were compared with the Mann–Whitney U-test. The overall survivals in OS patients with or without metastasis were compared using Kaplan–Meier survival curves and log-rank tests. Pearson’s Chi-squared test was used to analyze the correlation between *miR-24* expression in human OS tissues and clinicopathological parameters of OS patients.

## Results

### The expression of vWF was significantly increased in OS tissues and cell lines

**Figure 1:**
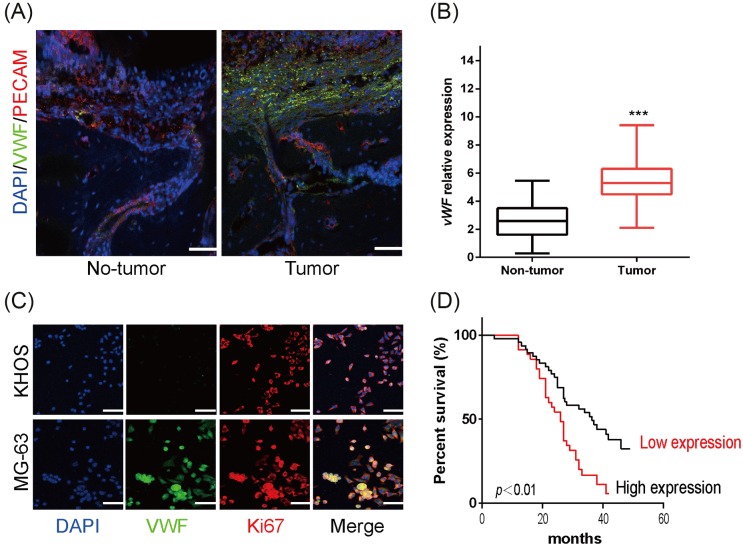
Expression pattern of vWF in OS tissues and cell lines (**A**) Representative immunofluorescence labeling images of vWF expression in human OS tissues (tumor) and adjacent non-cancerous tissues (non-tumor). vWF was shown in green, DAPI in blue, ECs were labeled by PECAM (red). Scale bars: 50 μm. (**B**) The relative expressions of vWF mRNA in human OS tissues (*n*=84) and adjacent non-cancerous tissues (*n*=84) were compared with the Mann–Whitney U-test. ***P<0.001. (**C**) Representative immunofluorescence labeling images of vWF expression in human OS cells MG-63 and KHOS cells. vWF was shown in green, DAPI in blue, Ki67 in red. Scale bars: 25 μm. (**D**) The overall survivals in OS patients with high or low vWF expression were compared using Kaplan–Meier survival curves and log-rank tests., ***P<0.001.

To uncover the role of vWF in the progression of OS, the expressions of vWF were analyzed in 84 human OS tissues by immunofluorescence labeling and quantitative real-time PCR (qRT-PCR) analysis. As is shown in Figure 1A, vWF was abundant around the cancer cells and ECs, a major source of vWF, in human OS tissues (tumor). Whereas, vWF was only found around EC in the adjacent non-cancerous tissues (non-tumor). Moreover, qRT-PCR analysis revealed that the relative expression of vWF mRNA in human OS tissues (*n*=84) significantly higher than that of paired non-cancerous tissues (*n*=84) (Figure 1B). Similar results were observed in OS cell lines. MG-63 cells had higher expression of vWF than that in vWF-deficit KHOS cells (Figure 1C). Moreover, Kaplan–Meier survival analysis demonstrated that higher vWF expression showed a poor survival in OS patients (Figure 1D).

### Correlation of *miR-24* with overall survival and clinicopathological parameters of OS patients

**Figure 2:**
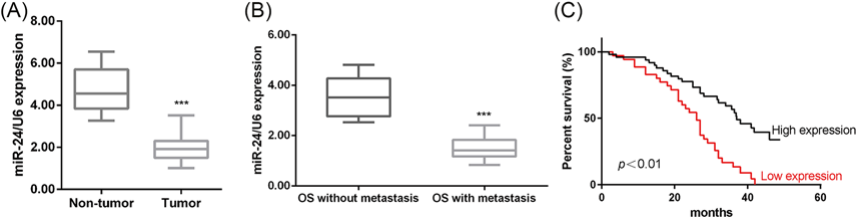
Expression of *miR-24* and its correlation with overall survival in OS patients (**A**) The relative expressions of *miR-24* in human OS tissues (tumor) (*n*=84) and adjacent non-cancerous tissues (non-tumor) (*n*=84) were compared with the Mann–Whitney U-test. U6 was used as the internal control. ***P<0.001, compared with non-tumor. (**B**) Comparison of the relative expression of *miR-24* between the metastatic OS tissues (*n*=47) and primary OS tissues (*n*=37). ***P<0.001, compared with OS without metastasis. (**C**) The overall survivals in OS patients with high or low *miR-24* expression were compared using Kaplan–Meier survival curves and log-rank tests.

Next, we analyzed the expressions of *miR-24* in OS. We found that the relative expressions of *miR-24* were markedly down-regulated in human OS tissues (Tumor) (*n*=84) (Figure 2A). To verify the functional involvement of *miR-24* in OS, the correlation between *miR-24* expression and clinicopathological parameters of OS patients (*n*=84) was analyzed. The relative expressions of *miR-24* in 84 OS tissues were examined by qRT-PCR. And, the median value of all 84 OS samples was chosen as the cut-off point for separating tumors with low-level expression of *miR-24* from high-level expression *miR-24* tumors. Statistical analysis confirmed that low *miR-24* expression in human OS tissues was significantly associated with tumor node metastasis and metastasis (Table 1). Accordingly, we noticed a lower level of *miR-24* in the metastatic OS tissues (*n*=47) compared with primary OS tissues (*n*=37) (Figure 2B). And further Kaplan–Meier survival analysis demonstrated that low *miR-24* expression predicted a poor survival in OS patients (Figure 2C). Together, these results revealed a potential role of *miR-24* in OS progression.

**Table 1:** Correlation between *miR-24* expression in human OS tissues and clinicopathological parameters of OS patients

Variables	*miR-24*			*X*^2^ value	*P* value
	All cases (*n*=84)	High expression (*n*=42)	Low expression (*n*=42)		
Age (years)				0.194	0.659
>60	36	17 (47.22%)	19 (52.78%)		
≤60	48	25 (52.08%)	23 (47.92%)		
Sex				0.426	0.514
Male	66	34 (51.52%)	32 (48.48%)		
Female	18	8 (44.44%)	10 (55.56%)		
Tumor size				0.202	0.653
≤5	32	15 (46.88%)	17 (53.12%)		
>5	52	27 (51.92%)	25 (48.08%)		
TNM stage				15.570	0.000
I+II	38	28 (73.68%)	10 (26.32%)		
III+IV	46	14 (30.43%)	32 (69.57%)		
Metastasis				13.960	0.000
Yes	47	15 (31.91%)	32 (68.09%)		
No	37	27 (72.97%)	10 (27.03%)		

Pearson’s Chi-squared test (*P<0.05). Abbreviation: TNM, tumor node metastasis.

### vWF was a novel target gene of *miR-24* in OS

**Figure 3:**
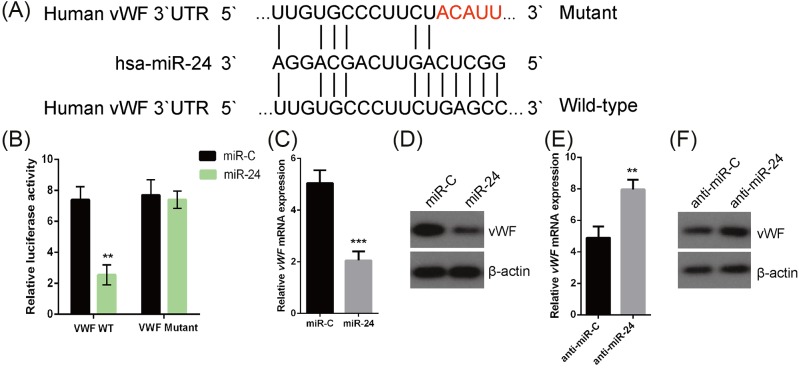
vWF was a target of *miR-24* in OS (**A**) According to the putative binding site of *miR-24* in the 3′-UTR of vWF (wild type), the mutated vWF 3′-UTR (mutant) was generated. (**B**) Dual luciferase reporter assay was performed in the OS cells MG-63 co-transfected with wild type/mutated vWF 3′-UTR reporters and *miR-24* mimic (*miR-24*) or its control, miR-C. **P<0.01, compared with miR-C. OS cells MG-63 were transfected with *miR-24* mimic or miR-C, and the level of (**C**) vWF mRNA and (**D**) protein was determined using qRT-PCR and Western blot analysis, respectively. ***P<0.001, compared with miR-C. The effect of *miR-24* inhibition on (**E**) vWF mRNA and (**F**) protein expression was also evaluated in the OS cells MG-63 transfected *miR-24* inhibitor (anti-*miR-24*) or its control, anti-miR-C. **P<0.01, compared with anti-miR-C.

Interestingly, qRT-PCR analysis showed a conspicuous negative correlation between vWF mRNA and *miR-24* expression in human primary OS tissues (Table 2). And consistent with previous report [21], the bioinformatics analysis revealed that *miR-24* interacted with the 3′-UTR of vWF in current study (Figure 3A). To further verify the interaction between *miR-24* and vWF3′UTR, the pmirGLO luciferase vectors containing *miR-24*-binding site of vWF 3′UTR (vWF WT) or a mutant miR-24-binding site (vWF mutant) were constructed. Luciferase report assay showed that *miR-24* overexpression markedly reduced the relative luciferase activity in the cells transfected with WT vWF, whereas *miR-24* expression had no significant effect on the relative luciferase activity of cells transfected with vWF mutant, (Figure 3B). We further determined the response of vWF expression to *miR-24* alternations. As shown in Figure 3C,D, *miR-24* overexpression caused significant down-regulation of vWF mRNA and protein. Conversely, silencing of *miR-24* resulted in a significant increase in vWF expression (Figure 3E,F). Collectively, these results supported the notion that vWF was a target gene of *miR-24* in OS MG-63 cells.

**Table 2:** The expression relationship of *miR-24* and vWF mRNA in human OS tissues

Variables	*miR-24*			*X*^2^ value	*P value
	Cases	High	Low		
vWF mRNA				19.091	0.000
High	42	12 (28.57%)	30 (71.43%)		
Low	42	32 (76.19%)	10 (23.81%)		

Pearson’s correlation (*P<0.05).

### *miR-24* suppressed OS cell proliferation, colony formation, and migration through inhibiting vWF

**Figure 4:**
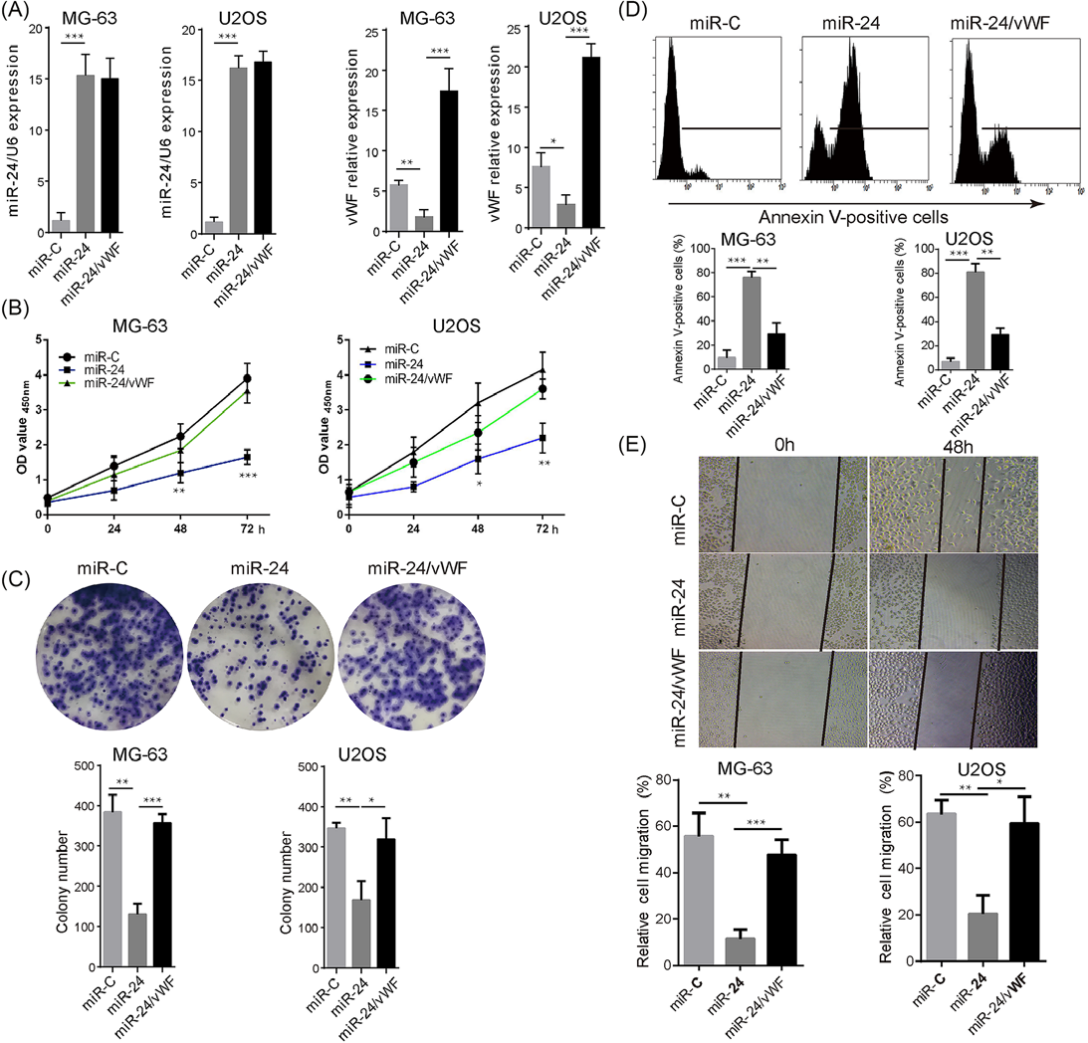
*miR-24* suppressed OS cell proliferation, migration, and colony formation through inhibiting vWF MG-63 and U2OS cells were transfected with *miR-24* mimic, miR-C, or *miR-24* mimic and pcDNA-vWF (vWF), respectively. (**A**) The expressions of *miR-24* and vWF mRNA were determined using qRT-PCR analysis. (**B**) The cell proliferation was assessed using CCK-8 assay. (**C**) Crystal violet staining was performed to detect the cell colony formation ability, and representative images were showed. (**D**) the cell apoptosis was assessed using Annexin V staining by flow cytometry. (**E**) The migration of MG-63 and U2OS cells was evaluated by wound-healing assay at 48 h after wound formation. Representative images and the cell counting results of MG-63 were showed. *P<0.05, **P<0.01, ***P<0.001.

**Figure 5:**
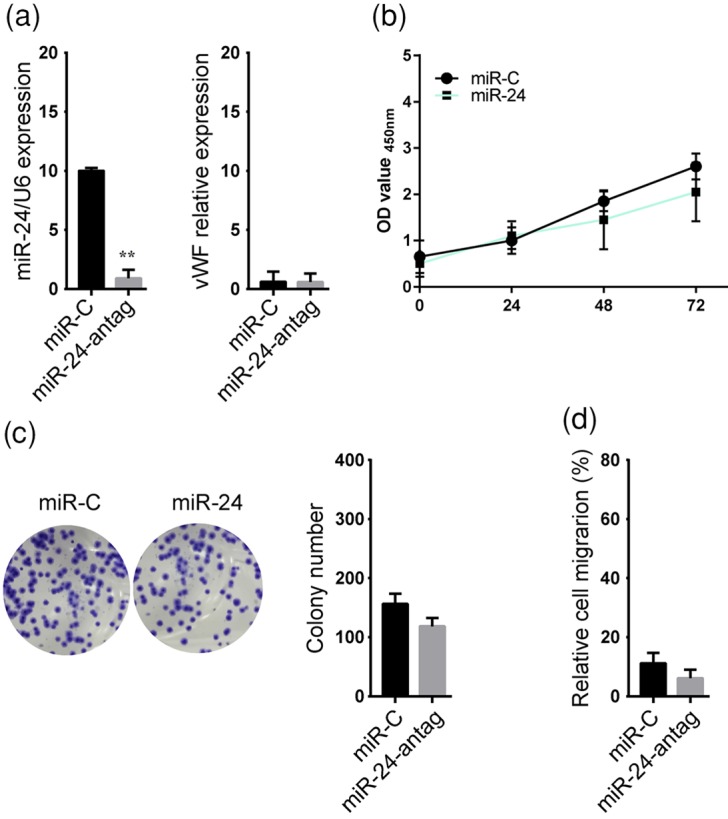
*miR-24* failed to suppress vWF-deficit OS cell proliferation, migration, and colony formation KHOS cells were transfected with miR-C or *miR-24* antagonist. (**A**) The expressions of *miR-24* and vWF mRNA were determined using qRT-PCR analysis in cells, respectively. (**B**) The cell proliferation was assessed using CCK-8 assay. (**C**) Crystal violet staining was performed to detect the cell colony formation ability, and representative images were showed. (**D**) The migration of KHOS cells was evaluated by wound-healing assay at 48 h after wound formation. **P<0.01.

Finally, we further dissected the physiological significance of the interaction between vWF and *miR-24* in OS using loss- and gain-of-function approaches. Overexpression of *miR-24* significantly repressed the expression of vWF in MG-63 and U2OS. Although did not significantly alter *miR-24* level, pcDNA-vWF transfection increased the expression of vWF mRNA in *miR-24* overexpressed MG-63 cells and U2OS cells, respectively (Figure 4A). In addition, co-overexpression of *miR-24* and vWF reversed, at least in part, the *miR-24*-mediated decrease in the proliferation and colony formation, and increase in cell apoptosis (Figure 4B–D). Consistently, *miR-24* notably inhibited the migration of MG-63 cells and U2OS cells, which could be attenuated through by co-overexpression of vWF (Figure 4E). Next, we performed the experiments to investigate the effects of *miR-24* on vWF-deficit KHOS cells. We found that inhibition of *miR-24* had limited effects on vWF level in KHOS cells (Figure 5A). Moreover, the inhibition of *miR-24* failed to suppress vWF-deficit KHOS cell proliferation (Figure 5B), migration (Figure 5C), and colony formation (Figure 5D). These results indicate that vWF, as a downstream effector of *miR-24*, plays an important role in OS progression.

## Discussion

vWF is a mainly expressed in ECs and megakaryocytes. vWF has important roles in hemostasis through facilitating platelet aggregation [22]. Additionally, studies have reported that plasma vWF was significantly increased in patients with colon cancer, ovarian cancer, and gastric adenocarcinoma [23, 24]. And high levels of plasma vWF were shown to be associated with cancer malignancy degree, overall survival rate, and poor prognosis [24]. Contradictorily, vWF was also reported to be involved in bone cancer cell apoptosis by inducing the binding of osteoprotegerin to TRAIL [13], implying a multiple facet of vWF in cancer progression. Recently, vWF was detected also in cancer cells of non-endothelial origin including OS [25]. Consistent with these results, vWF was found to be significantly up-regulated in metastatic OS tissues compared with primary OS tissues and associated with increased metastasis [14]. Moreover, vWF was expressed in OS cell line SAOS2 and increased the adhesion capacity of SAOS2 cells to endothelial monolayer, causing increased cancer cell extravasation and transmigration [26]. Therefore, target vWF attenuated the metastatic characteristics of OS cells, representing a potential target of OS therapy. The current study provided new evidence that vWF was abundant in human primary OS tissues.

More importantly, in human primary OS tissues, our study showed a negative correlation between the expression of vWF mRNA and *miR-24*, which has been demonstrated to greatly down-regulate in OS cells and to inhibit OS metastasis [21]. Accumulating studies indicated that miRNAs might serve as a potential prognostic predictor and/or therapeutic target in the tumorigenesis and progression of OS [27]. *miR-24*, which is abnormally expressed in various cancer types, has also been reported as one of the candidates in OS. Previous reports of dysregulated *miR-24* in OS showed that *miR-24* was able to inhibit the proliferation, invasion, and migration of OS cells, suggesting *miR-24* as a tumor suppressor in OS progression [20, 21]. This conclusion was further strengthened by our findings that *miR-24* was markedly down-regulated in the metastatic OS tissues compared with primary OS tissues and suppressed OS cell proliferation, colony formation, and migration. *miR-24* was identified as an independent prognostic marker for acute leukemia, colorectal cancer, aflatoxin B1-related hepatocellular carcinoma, and human tongue squamous cell carcinoma [28–31]. In the present study, the clinical data showed that low *miR-24* expression in human OS tissues was significantly associated with tumor metastasis and predicted a poor survival in OS patients, thus provided new insight into the clinical significance of *miR-24* expression in OS patients.

miRNAs interacting with the 3′-UTR of mRNA constitute a transcription or post-transcription regulation mechanisms involved in various biological functions and the pathogenesis of diseases. The previous study has reported that vWF 3′-UTR as a target of *miR-24* based on the bioinformatics analysis [22], the present data also evidenced that vWF was a target gene of *miR-24* in OS as shown by that *miR-24* mimic inhibited, whereas *miR-24* inhibitor increased, both the mRNA and protein levels of vWF in OS cells. Furthermore, it was noticed that vWF overexpression predominantly attenuated the *miR-24*-mediated inhibition on the proliferation and migration of OS cells without altering the endogenous *miR-24* level. Thus, our data indicated that vWF dysregulation was the critical mediator that contributed to tumor suppression induced by *miR-24*.

In summary, we demonstrated that increased vWF by *miR-24* down-regulation promoted the proliferation and migration of OS cells and further identified the clinical significance of *miR-24* expression in OS patients. Although additional prospective analysis is needed in other OS cell lines and *in vivo*, the present data confirmed the importance of *miR-24*/vWF signaling axis in OS progression and provided a new mechanistic insight into the functional involvement of vWF in OS in addition to its clinical observations.

## Abbreviations

CCK-8: cell counting kit-8
EC: endothelial cell
OS: osteosarcoma
PVDF: polyvinylidene fluoride
vWF: von Willebrand factor

## References

[1] Lin Y.H., Jewell B.E., Gingold J., Lu L., Zhao R., Wang L.L. (2017) Osteosarcoma: molecular pathogenesis and iPSC modeling. Trends Mol. Med. 23, 737–755 10.1016/j.molmed.2017.06.004 28735817 PMC5558609

[2] Harrison D.J., Geller D.S., Gill J.D., Lewis V.O. and Gorlick R. (2018) Current and future therapeutic approaches for osteosarcoma. Expert Rev. Anticancer Ther. 18, 39–50 10.1080/14737140.2018.1413939 29210294

[3] Lof A., Muller J.P. and Brehm M.A. (2018) A biophysical view on von Willebrand factor activation. J. Cell. Physiol. 233, 799–810 10.1002/jcp.25887 28256724

[4] Bauer A.T., Suckau J., Frank K., Desch A., Goertz L., Wagner A.H. (2015) von Willebrand factor fibers promote cancer-associated platelet aggregation in malignant melanoma of mice and humans. Blood 125, 3153–3163 10.1182/blood-2014-08-595686 25977583 PMC4432010

[5] Yang X., Sun HJ., Li ZR., Zhang H., Yang WJ., Ni B. (2015) Gastric cancer-associated enhancement of von Willebrand factor is regulated by vascular endothelial growth factor and related to disease severity. BMC Cancer 15, 80 10.1186/s12885-015-1083-6 25886574 PMC4340498

[6] Franchini M., Frattini F., Crestani S., Bonfanti C. and Lippi G. (2013) von Willebrand factor and cancer: a renewed interest. Thromb. Res. 131, 290–292 10.1016/j.thromres.2013.01.015 23394808

[7] Morganti M., Carpi A., Amo-Takyi B., Sagripanti A., Nicolini A., Giardino R. (2000) Von Willebrand’s factor mediates the adherence of human tumoral cells to human endothelial cells and ticlopidine interferes with this effect. Biomed. Pharmacother. 54, 431–436 10.1016/S0753-3322(00)00006-8 11100896

[8] Karpatkin S., Pearlstein E., Ambrogio C. and Coller B.S. (1988) Role of adhesive proteins in platelet tumor interaction *in vitro* and metastasis formation *in vivo*. J. Clin. Invest. 81, 1012–1019 10.1172/JCI113411 3280598 PMC329625

[9] Marfia G., Navone S.E., Fanizzi C., Tabano S., Pesenti C., Abdel Hadi L. (2016) Prognostic value of preoperative von Willebrand factor plasma levels in patients with Glioblastoma. Cancer Med. 5, 1783–1790 10.1002/cam4.747 27236861 PMC4887291

[10] El Sherif N.H., Narouz M.F., Elkerdany T.A. and El Habashy S.A. (2014) Von Willebrand factor and factor VIII levels in Egyptian children with newly diagnosed acute lymphoblastic leukemia in relation to peripheral blast cells and steroid therapy. J. Pediatr. Hematol. Oncol. 36, 518–523 10.1097/MPH.0000000000000219 25105915

[11] Garam N., Malati E., Sinkovits G., Gombos T., Szederjesi A., Barabas L. (2018) Platelet count, ADAMTS13 activity, von Willebrand factor level and survival in patients with colorectal cancer: 5-year follow-up study. Thromb. Haemost. 118, 123–131 10.1160/TH17-07-0548 29304532

[12] Zietek Z., Iwan-Zietek I., Paczulski R., Kotschy M. and Wolski Z. (1996) von Willebrand factor antigen in blood plasma of patients with urinary bladder carcinoma. Thromb. Res. 83, 399–402 10.1016/0049-3848(96)00149-1 8873348

[13] Baud’huin M., Duplomb L., Téletchéa S., Charrier C., Maillasson M., Fouassier M. (2009) Factor VIII-von Willebrand factor complex inhibits osteoclastogenesis and controls cell survival. J. Biol. Chem. 284, 31704–31713 10.1074/jbc.M109.030312 19758994 PMC2797241

[14] Eppert K., Wunder J.S., Aneliunas V., Kandel R. and Andrulis I.L. (2005) von Willebrand factor expression in osteosarcoma metastasis. Mod. Pathol. 18, 388–397 10.1038/modpathol.3800265 15467717

[15] Sayed D. and Abdellatif M. (2011) MicroRNAs in development and disease. Physiol. Rev. 91, 827–887 10.1152/physrev.00006.2010 21742789

[16] Rodriguez A.S., Engel T., Palfi A., Farrar G.J., Henshall D.C. and Jimenez-Mateos E.M. (2017) Tubby-like protein 1 (Tulp1) is a target of microRNA-134 and is down-regulated in experimental epilepsy. Int. J. Physiol. Pathophysiol. Pharmacol. 9, 178–187 29348794 PMC5770514

[17] Bartel D.P. and Chen C.Z. (2004) Micromanagers of gene expression: the potentially widespread influence of metazoan microRNAs. Nat. Rev. Genet. 5, 396–400 10.1038/nrg1328 15143321

[18] Yan L., Ma J., Zhu Y., Zan J., Wang Z., Ling L. (2018) miR-24-3p promotes cell migration and proliferation in lung cancer by targeting SOX7. J. Cell Biochem. 119, 3989–3998 10.1002/jcb.26553 29231262

[19] Song L., Yang J., Duan P., Xu J., Luo X., Luo F. (2013) MicroRNA-24 inhibits osteosarcoma cell proliferation both *in vitro* and *in vivo* by targeting LPAATbeta. Arch. Biochem. Biophys. 535, 128–135 10.1016/j.abb.2013.04.001 23578572

[20] Liu Z., Liu Z., Zhang Y., Li Y., Liu B. and Zhang K. (2017) miR-24 represses metastasis of human osteosarcoma cells by targeting Ack1 via AKT/MMPs pathway. Biochem. Biophys. Res. Commun. 486, 211–217 10.1016/j.bbrc.2017.02.045 28189676

[21] Xiang Y., Cheng J., Wang D., Hu X., Xie Y., Stitham J. (2015) Hyperglycemia repression of miR-24 coordinately upregulates endothelial cell expression and secretion of von Willebrand factor. Blood 125, 3377–3387 10.1182/blood-2015-01-620278 25814526 PMC4447857

[22] Hassan M.I., Saxena A. and Ahmad F. (2012) Structure and function of von Willebrand factor. Blood Coagul. Fibrinolysis 23, 11–22 10.1097/MBC.0b013e32834cb35d 22089939

[23] Yang A.J., Wang M., Wang Y., Cai W., Li Q., Zhao T.T. (2018) Cancer cell-derived von Willebrand factor enhanced metastasis of gastric adenocarcinoma. Oncogenesis 7, 12 10.1038/s41389-017-0023-5 29362409 PMC5833464

[24] Terraube V., Marx I. and Denis C.V. (2007) Role of von Willebrand factor in tumor metastasis. Thromb. Res. 120, S64–S70 10.1016/S0049-3848(07)70132-9 18023715

[25] Mojiri A., Stoletov K., Carrillo M.A., Willetts L., Jain S., Godbout R. (2017) Functional assessment of von Willebrand factor expression by cancer cells of non-endothelial origin. Oncotarget 8, 13015–13029 10.18632/oncotarget.14273 28035064 PMC5355073

[26] Ji X., Wang E. and Tian F. (2018) MicroRNA-140 suppresses osteosarcoma tumor growth by enhancing anti-tumor immune response and blocking mTOR signaling. Biochem. Biophys. Res. Commun. 495, 1342–1348 10.1016/j.bbrc.2017.11.120 29170130

[27] Organista-Nava J., Gomez-Gomez Y., Illades-Aguiar B., Del Carmen Alarcon-Romero L., Saavedra-Herrera M.V., Rivera-Ramirez A.B. (2015) High miR-24 expression is associated with risk of relapse and poor survival in acute leukemia. Oncol. Rep. 33, 1639–1649 10.3892/or.2015.3787 25672522 PMC4358084

[28] Gao Y., Liu Y., Du L., Li J., Qu A., Zhang X. (2015) Down-regulation of miR-24-3p in colorectal cancer is associated with malignant behavior. Med. Oncol. 32, 362 10.1007/s12032-014-0362-4 25502080

[29] Liu Y.X. and Long X.D. (2014) MicroRNA-24 modulates aflatoxin B1-related hepatocellular carcinoma prognosis and tumorigenesis. BioMed. Res. Int. 2014, 482926 24800232 10.1155/2014/482926 PMC3997078

[30] Zheng X., Li J., Peng C., Zhao J., Chi J., Meng X. (2015) MicroRNA-24 induces cisplatin resistance by targeting PTEN in human tongue squamous cell carcinoma. Oral Oncol. 51, 998–1003 10.1016/j.oraloncology.2015.08.002 26365986

[31] Slaby O., Laga R. and Sedlacek O. (2017) Therapeutic targeting of non-coding RNAs in cancer. Biochem. J. 474, 4219–4251 10.1042/BCJ20170079 29242381

