# Expression of Concern: von Willebrand factor rescued by miR-24 inhibition facilitates the proliferation and migration of osteosarcoma cells *in vitro*

**DOI:** 10.1042/BSR-20180372_EOC

**Published:** 2020-07-29

**Keywords:** migration, miR-24, osteosarcoma, proliferation, von Willebrand factor

The Editorial Office has been made aware of potential issues surrounding the scientific validity of this paper, hence has issued an expression of concern to notify readers whilst the Editorial Office investigates.

